# Effect of a honey-sweetened beverage on muscle soreness and recovery of performance after exercise-induced muscle damage in strength-trained females

**DOI:** 10.3389/fphys.2024.1426872

**Published:** 2024-09-17

**Authors:** Hadis Hemmati, Walaa Jumah Alkasasbeh, Mohammad Hemmatinafar, Mohsen Salesi, Sepideh Pirmohammadi, Babak Imanian, Rasoul Rezaei

**Affiliations:** ^1^ Department of Sport Sciences, Faculty of Education and Psychology, Shiraz University, Shiraz, Iran; ^2^ Program of Sports Management and Training, Department of Administration and Curriculum, Faculty of Arts and Educational Sciences, Middle East University, Amman, Jordan

**Keywords:** honey-sweetened beverage, DOMS, recovery, female athletes, strength performance

## Abstract

**Background:**

This study explores a novel approach to mitigating delayed-onset muscle soreness (DOMS), a common issue among strength-trained females. By investigating the potential of a honey-sweetened beverage, which contains anti-inflammatory properties, we aim to enhance muscle recovery after exercise-induced muscle damage (EIMD).

**Method:**

A randomized, cross-over, placebo-controlled, and double-blinded study was conducted with sixteen female strength athletes. Firstly, the baseline measurements were recorded, and participants were randomly divided into two conditions: honey-sweetened beverage (HSB; 70 g of honey in 250 mL water with a concentration of 28%) and placebo (PLA; 250 mL of water with 70 g of artificial sweetener). The HSB and PLA were consumed 90 min before the EIMD started (200 vertical jumps with 10% body-weighted vests). Recovery monitoring of performance indicators and DOMS was performed after EIMD. The results of wall-sit, V-Sit and reach flexibility test (VSFT), vertical jump height (VJH), pressure pain threshold (PPT), and one repetition maximum (IRM) tests were recorded 48 h after EIMD. Also, DOMS was recorded using the visual analog scale (VAS) before the start of the test and immediately, 12, 24, and 48 h after EIMD. A one-week interval was considered a washout period for each condition. The collected data were analyzed by repeated measures of ANOVA and Bonferroni *post hoc* test and dependent t-test at *P* ≤ 0.05 level.

**Results:**

Compared to PLA, HSB ingestion improves wall-sit performance (*p* = 0.003), 1RM (*p* = 0.019), and RPE (*p* = 0.003) after EIMD. However, no significant differences were observed between BL, PLA, and HSB in VJH (*p* = 0.384), VSFT (*p* = 0.840), and PPT (*p* = 0.151) after EIMD. Furthermore, HSB, compared to PLA, considerably decreased the values of DOMS immediately, 12, 24, and 48 h after EIMD (*p* < 0.05).

**Conclusion:**

Our findings illustrated that ingesting HSB in strength-trained females can be a helpful strategy for improving recovery indicators such as muscle strength, endurance, and muscle soreness after EIMD.

## Introduction

Sports activities are essential to promote adaptation and improve sports performance. However, sports involving intense, unusual, and/or eccentric activities significantly stress skeletal muscle, leading to exercise-induced muscle damage (EIMD). EIMD impairs several measures of athletic performance, such as maximal power output, speed and agility, jumping ability, and isometric and isokinetic strength (Burt et al., 2023). Delayed onset muscle soreness (DOMS) describes an ultrastructural muscle injury and is defined as pain, muscle stiffness, swelling, loss of force-generating capacity, decreased joint range of motion, and decreased proprioceptive function (Sonkodi, 2022). Also, DOMS is a consequence often associated with EIMD (Hill et al., 2014). Unaccustomed or intense exercise activities that involve unusual contractions cause DOMS with neuromuscular changes lasting several days (Sonkodi, 2022). Although DOMS is classified as a type of mild muscle damage, it is one of the most common causes of compromised athletic performance (Heiss et al., 2019). The mechanism of DOMS is still not fully understood. Still, several theories, such as lactic acid, muscle spasm, inflammation, connective tissue damage, muscle damage, enzyme flow, and recently nerve damage and micro-damage theory, have tried to explain the mechanism of DOMS (Sonkodi et al., 2020; Sonkodi et al., 2021). However, none of these theories or agents have fully explored the mechanism of DOMS. It is important to note that DOMS differs from pain experienced during or immediately after exercise because DOMS can occur without muscle damage (Hayashi et al., 2017).

Nutritional supplements are widely used as a nutritional strategy to improve and maintain performance in exercise (Close et al., 2016). EIMD is characterized by a primary response from mechanical stress during exercise and a secondary inflammatory response (Clarkson et al., 1992; Tiidus, 1998). Therefore, nutritional supplements with anti-inflammatory and antioxidant properties can potentially reduce muscle damage (Tanabe et al., 2021). Honey is a natural source of energy that, due to its high viscosity and sweet taste, has several protective and therapeutic effects on physiological disorders such as skin, certain nervous, ailments, measles, period pains, and postnatal disorders (Eteraf-Oskouei and Najafi, 2013; Bansal et al., 2005; Meda et al., 2004). Polyphenols in honey play a central role in preventing and managing several diseases (Hossen et al., 2017). These compounds have beneficial indications for having antioxidant, anti-inflammatory, anti-carcinogenic effects and broader protective activities (Samarghandian et al., 2017a). Flavonoids and phenolic acids are the leading group of phenolic compounds in honey (Cheung et al., 2019). The anti-inflammatory properties of honey can be attributed to its phenolic and flavonoid compounds, while other sugar and non-sugar substances have immunomodulatory effects (Samarghandian et al., 2017b). A large number of *in vivo* and *in vitro* studies have identified the potential anti-inflammatory mechanisms of honey and its compounds, which include downregulation of NF-KB and MAPK signaling pathways, leading to decreased levels of proinflammatory cytokines, including tumor necrosis factor α (TNF-α), interleukin-6 (IL-6) and interleukin-1β (IL-1β) (Ahmed et al., 2018). It has been shown that honey may improve physical performance. It may also reduce fatigue and inflammation caused by sedentary activity. The Low GI and anti-fatigue properties of honey can cause physiological adaptations in sports activities among people (Ali et al., 2021).

Therefore, consuming supplements containing honey for health may increase when combined with sports exercises (Ali et al., 2021). In this regard, research has shown that the consumption of honey can improve the physical fitness of athletes (Hasan et al., 2016). Furthermore, researching the effects of consuming honey solution on the levels of injury-related factors after three semi-competitive kickboxing matches in men showed that the amounts of lactate, creatine kinase, and lactate dehydrogenase (60 and 120 min) in the honey group were significantly reduced compared to the placebo (Esmaeelzadeh et al., 2022). Due to the performance and antioxidant properties of honey, as well as its availability, considering that a few researches have been done on honey consumption and muscle soreness by examining the indicators of functional companions, and contradictory results have been observed in them; therefore, this study investigated the effect of ingesting a honey-sweetened beverage on muscle soreness and performance recovery after EIMD in strength-trained females.

## Methodology

### Participants

Sixteen women with an average of 5 years of bodybuilding experience (Age: 27 ± 4 years, Height: 163 ± 4 cm, Weight: 59 ± 7 kg, and BMI: 21 ± 2 kg/m^2^) participated voluntarily in this study. Participants had no known diseases or medical issues, no history of allergy to honey, and were not consuming any supplements or medications. Furthermore, the participants did not smoke or consume alcoholic beverages at the time of data collection. Before the implementation of the intervention, the study procedures were explained to the participants, and written consent was obtained. This study was reviewed and approved by the Research Ethics Committees of the Faculty of Psychology and Educational Sciences, Shiraz University, Shiraz, Iran (ethics approval code: SEP.14023.48.5571, 2023) and in accordance with the principles in the Declaration of Helsinki.

### Study design

**FIGURE 1:**
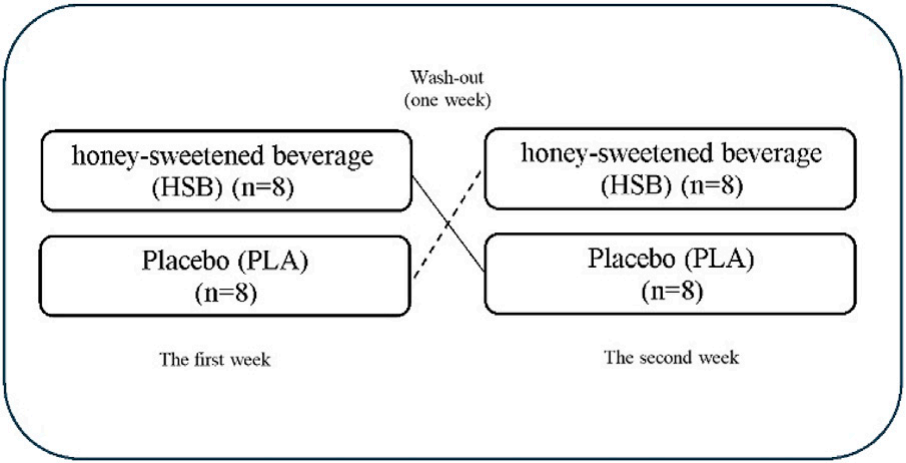
Cross-over and double-blinded study design in two conditions.

**FIGURE 2:**
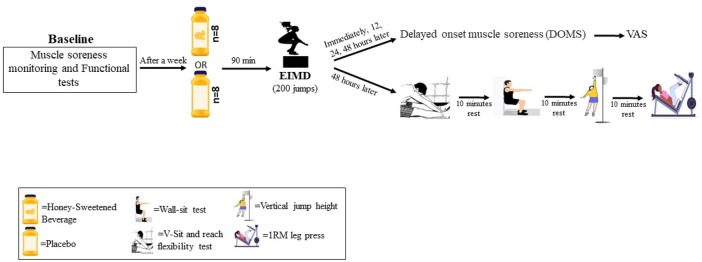
The protocol of taking supplements and performing tests.

This study was randomized, cross-over, placebo-controlled, and double-blinded, as illustrated in Figures 1, 2. Before the investigation, all participants attended a familiarization session to learn about the testing protocols and procedures. During the familiarization session, the wall-sit, V sit and reach (VSFT), vertical jump height (VJH), pressure pain threshold (PPT), and one repetition maximum (IRM) tests were recorded for the baseline measurement (BL). They were then divided into two conditions: the honey-sweetened beverage (HSB) condition, which consumed 70 g of honey in 250 mL of water with a concentration of 28%, and the placebo (PLA) condition, which consumed 250 mL of water with 70 g of artificial sweetener (Stevia, Fibrelle, Turkey). Both conditions consumed their assigned drink 90 min before the EIMD (200 vertical jumps with 10% body-weighted vests). The results of the wall-sit, VSFT, VJH, PPT, and IRM tests were recorded 48 h after EIMD. Additionally, DOMS was measured using the visual analog scale (VAS) at baseline and immediately, 12, 24, and 48 h after EIMD. DOMS, a common symptom of EIMD, typically appears between 8 and 24 h after the muscle-damaging exercise, peaks between 24 and 48 h, and usually subsides within 96 h (Damas et al., 2016; Bongiovanni et al., 2020). Therefore, the special functional tests were conducted 48 h after EIMD in this study. Participants were given a list of routine dietary sources of antioxidants and caffeine and asked to avoid consuming them 24 h before each exercise test session. A one-week interval was considered a washout period for each condition (Figure 2). Previous research shows that the menstrual cycle can affect the research results (McNulty et al., 2020). For this purpose, to minimize the effects of hormonal changes that occur during the menstrual cycle on the measured variables, the EIMD, supplementation, and functional tests were carried out during the follicular phase. The menstrual cycle phase for each participant was determined using the Menstrual Cycle Questionnaire, and people who had similar periods at the test were selected (Matteson, 2017). Additionally, all participants were naturally menstruating and were not using contraceptive methods.

### Supplementation procedures

Based previous studies (Tartibian et al., 2016; Tartibian and Maleki, 2012) in the HSB condition, the participants drank a mixture of 70 g of honey and 250 mL of water with a concentration of 28% (Mehran Avishe Company, Abshan Honey, Iran). Biochemical tests revealed that honey’s composition includes fructose 40 g %, sucrose 4.9 g %, glucose 25 g %, acidity 13 g %, moisture 14 g %, vitamin E 0.8 mg, vitamin C 2.5 mg, vitamin A 0.51 mg, calcium 15 mg, selenium 0.55 mg, copper 0.099 mg, chromium 0.0015 mg, iron 0.709 mg, cobalt 0.022 mg, zinc 0.83 mg, and glutathione reductase 0.68 mg. The pH of the honey was measured at 3.6. In the placebo condition, the participants drank 250 mL of water with 70 g of artificial sweetener without any calories (Stevia, Fibrelle, Turkey). Both groups consumed their assigned drink 90 min before the EIMD. All the trials were completed around the same time, between 10 and 11 a.m. The participants were monitored for any side effects resulting from the interventions, and any new symptoms that appeared after starting the interventions were considered and recorded as side effects. No side effects were reported among participants following HSB and PLA supplementation. Participants were given the same breakfast containing 350–400 kcal, with 64% carbohydrates, 20% protein, and 16% fat, 2 h before the exercise test session (Pirmohammadi et al., 2023). Participants were instructed to maintain a regular diet throughout the testing period, avoid food an hour before testing, and avoid strenuous exercise 24 h before each trial. They were also provided with a list of dietary sources of caffeine and asked to refrain from consuming these 24 h before each exercise test session.

### Exercise-induced muscle damage protocol

Before the EIMD protocol, the participants performed a 10-min warm-up of dynamic movements, slow running, and stretching exercises. Thereafter, participants completed 200 vertical jumps with weighted vests (equivalent to 10% of body weight) to induce muscle damage. For this purpose, each person performed ten sets of 20 maximum jumps (one jump every 4 s). Two minutes of rest (sitting in a chair) were included between sets. To ensure the program’s rigidity, the participants presented and assessed the rate of perceived exertion (RPE) scale immediately after the test. The EIMD protocol was adapted from previous literature on dietary supplementation (Clifford et al., 2016; Clifford et al., 2017). During the study, all participants were members of the same training camp, and their training protocol was the same under the supervision of trainers.

### Examination of delayed onset muscle soreness by the VAS scale

The VAS scale measured the amounts of DOMS. On this scale, a horizontal line of 10 cm is drawn, at the beginning of which the phrase is painless and at the end of which the word is in severe pain. VAS is a number that allows a person to express the severity of their pain and is used in experimental and clinical studies. The subjects determined their perception of the severity of delayed onset muscle soreness before the start of the test, immediately, 12, 24, and 48 h after EIMD. By measuring the distance of points marked from the line’s origin with a ruler, the pain score of each person was recorded in millimeters (Tartibian et al., 2009).

### Rating of perceived exertion

The Borg 6–20 category ratio scale was initially explained to subjects during their familiarization trial, and instructions were repeated before subsequent trials. Participants are asked to rate their rating of perceived exertion (RPE) immediately after EIMD, combining all sensations and feelings of physical stress and fatigue. Also, they are told to disregard any one factor, such as leg pain or shortness of breath, but to try to focus on the whole feeling of exertion (Williams, 2017).

### Pressure pain threshold

The study determined the pressure pain threshold (PPT) using a blood pressure cuff at the midpoint of the femur (Blood Pressure Cuff - Thigh - Double Tube, MDF Instruments, United States). The participants were seated on a chair with their knees bent at a 90-degree angle. A 2.5 cm diameter and 25 cm length plastic tube was placed around the femur midline of the dominant leg. The blood pressure gauge cuff was then placed around the participant’s thigh and uniformly inflated. The investigator recorded the pressure level at the onset of pain as the PPT in mmHg (Hemmatinafar et al., 2023).

### V-sit and reach flexibility test (VSFT)

During the VSFT, participants sat shoeless on the floor with their soles 30 cm apart. A meterstick was placed between the participant’s legs so the 23 cm mark aligned with the participant’s heels (López-Miñarro et al., 2008). Subjects were later asked to place both hands together and extend forward as far as possible. The best of three attempts was recorded as the final score.

### Vertical jump height

Sargent’s jump test evaluated the Vertical Jump Height (VJH). The participants chalk the end of their fingertips. Then, stand beside the wall, the shortest distance from it, keeping both feet on the ground, reach up as high as possible with one hand, and mark the wall with the tips of the fingers (M1). From a static position, the participants jump as high as possible and mark the wall with the chalk on their fingers (M2). Then, the assistant recorded the distance between M1 and M2. The test is repeated three times, with a 1-min rest between each attempt. The best score is considered the final result (Pirmohammadi et al., 2023).

### Wall-sit test

The wall-sit test was used to evaluate lower-body muscle endurance. The correct posture was sitting, shoulder width straight and attached to the wall, knees at 90°, shoulders to the wall, and arms hanging straight down. For this test, the maximum time to exhaustion was defined as the time interval from the task’s start until any of these positions could not be maintained. Participants must do their best to maintain the correct position throughout the test while receiving no verbal encouragement (Lubans et al., 2011).

### One maximum repetition (1-RM)

The Brzycki equation determines the maximum strength of athletes. The 1-RM measurement test used a leg press to calculate this strength. This equation is used for sub-maximum repetitions (less than ten repetitions). To use this test, the person repeats the maximum weight shift to the limit of the weight, and then, according to the following equation, a maximum repeat is estimated for that movement [1-RM = Weight lifted ÷ (1.0278 − (0.0278 × Number of repetitions))] (Scott et al., 2016).

### Data analysis

The collected data was analyzed using descriptive and inferential statistical methods. The Kolmogorov-Smirnov test was used to determine the normality of the data distribution. As the data was found to be normally distributed, two-way (condition × time) repeated measures ANOVA and Bonferroni *post hoc* test were conducted to analyze the DOMS data, along with the repeated measures ANOVA and Bonferroni *post hoc* test for functional tests data and dependent t-test to analysis the RPE results. Hypothesis testing was also used to estimate the mean difference (MD) and 95% confidence interval (95% CI). Additionally, SPSS software (version 26, IBM-SPSS Inc., Chicago, IL, United States) was utilized to analyze the data. The level of statistical significance was considered to be *P* ≤ 0.05.

## Results

Descriptive characteristics (including mean and standard deviation) are reported in Table 1.

**TABLE 1:** The functional variables were measured in the two different experimental conditions.

Variable	Session 1	Session 2	MD	Sig	95% CI
	Mean ± SD	Mean ± SD			
VAS baseline (mm)	5.2 ± 0.6	5 ± 0.6	0.18	0.383	−0.25–0.63

	PLA	HSB	MD	Sig	95% CI
	Mean ± SD	Mean ± SD			
VAS-immediately (mm)	62.5 ± 4.2	46.5 ± 2.9	15.93	0.001	8.97–22.90
VAS-12 h (mm)	36.87 ± 2.9	25.3 ± 2.1	11.56	0.001	7.02–16.09
VAS-24 h (mm)	50 ± 3.3	35.6 ± 2.7	14.37	0.001	7.16–21.58
VAS-48 h (mm)	55 ± 3.3	37.8 ± 2.6	17.18	0.001	11.51–22.85
RPE	16.5 ± 1.5	14.8 ± 1	−1.75	0.001	−2.31–−1.18

PLA, placebo; HSB, honey-sweetened beverage.

**FIGURE 3:**
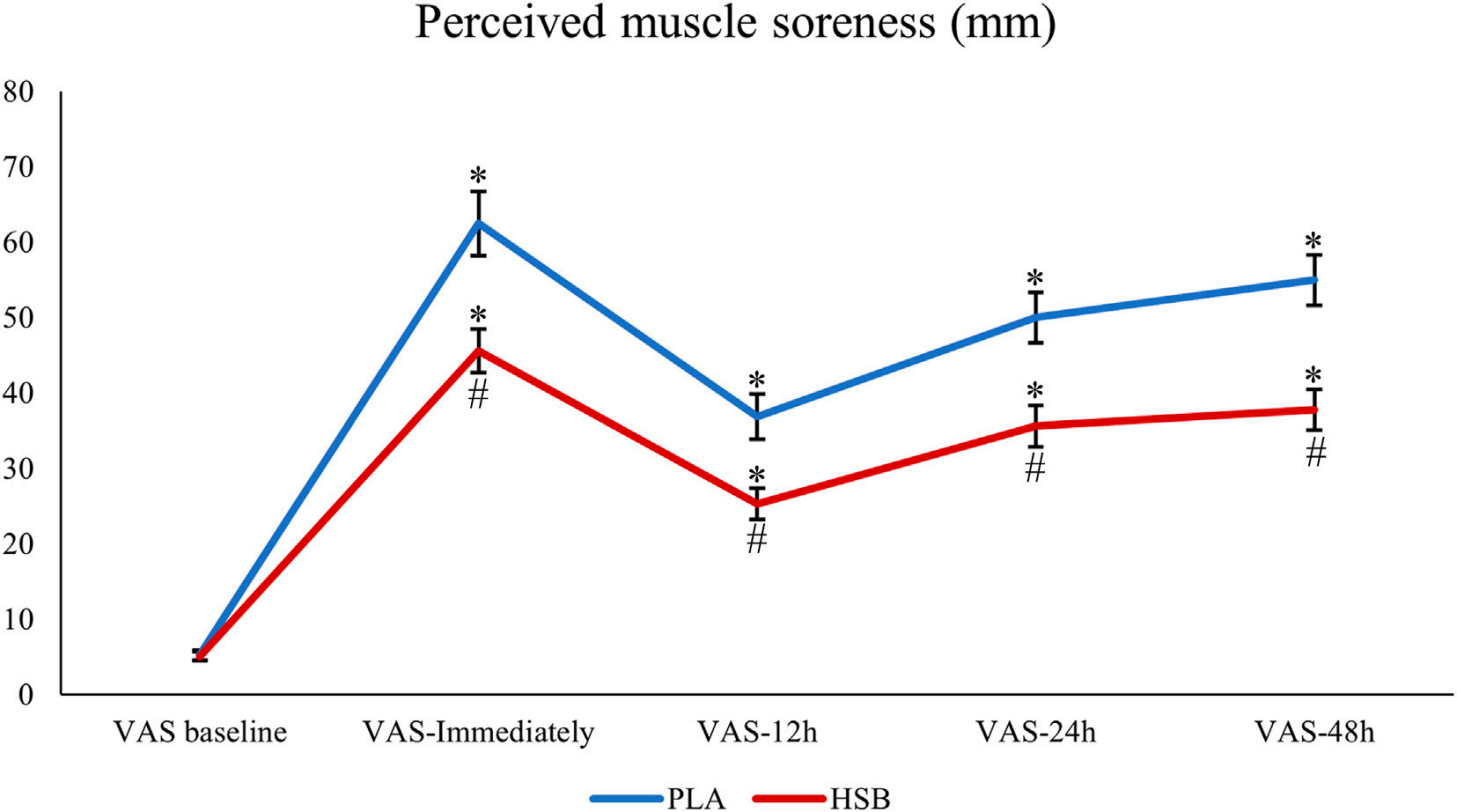
Perceived muscle soreness changes using the VAS scale in PLA or HSB conditions. (PLA, Placebo; HSB, Honey-Sweetened Beverage). *, Significant difference with VAS-baseline (*p* < 0.05). #, Significant difference between PLA and HSB in any conditions (*p* < 0.05).

**FIGURE 4:**
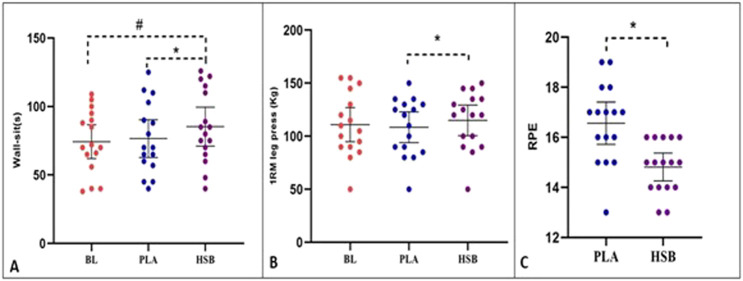
Changes in wall sit, 1RM leg press, and RPE in three conditions of this study (BL, Baseline; PLA, Placebo; HSB, Honey-Sweetened Beverage). *, Significant difference between PLA and HSB in any conditions (*p* < 0.05). #, Significant difference between HSB and BL in any conditions (*p* < 0.05).

The statistical analysis results using a two-way repeated measures ANOVA showed that the main effect of the condition [F = 46.76, *p <* 0.001], time [F = 114.52, *p <* 0.001], and interaction (condition × time) [F = 10.08, *p < 0*.*001*] was significant for DOMS. Further analysis using the Bonferroni test revealed a considerable increase in DOMS immediately, 12, 24, and 48 h after EIMD compared to baseline in both session 1 and session 2 (*p* < 0.05). Additionally, there was no difference in DOMS at baseline between session 1 and session 2 (*p* > 0.05). However, DOMS immediately, 12, 24, and 48 h after EIMD were significantly higher in PLA compared to HSB (*p* < 0.05). This information is also presented in Figure 3 and Table 1. Additionally, the results of the dependent t-test demonstrated a considerable decrease between the HSB and PLA conditions in RPE (MD = −1.75, *P* < 0.001, 95% CI [−2.31 – −1.18], Figure 4C; Table 1).

**FIGURE 5:**
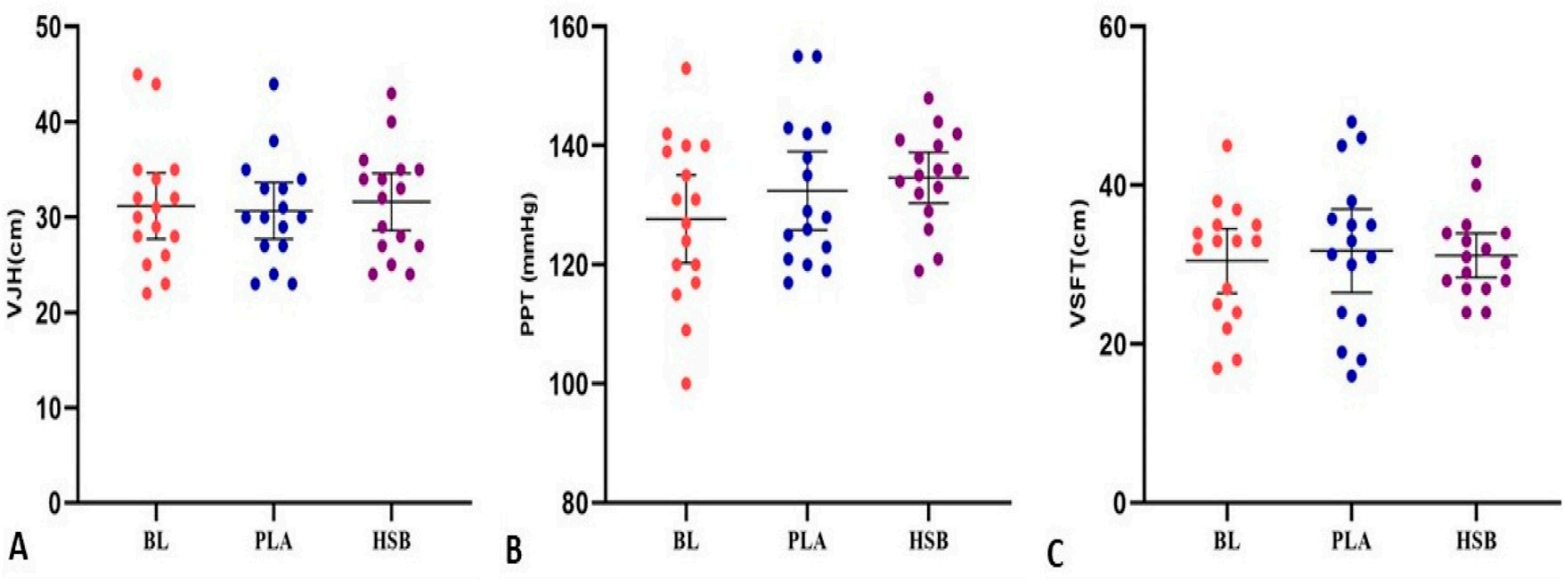
Changes in VJH (Vertical jump height), PPT (Pressure pain threshold), and VSFT (V sit and reach) in three conditions of this study (BL, Baseline; PLA, Placebo; HSB, Honey-Sweetened Beverage).

The repeated measure ANOVA test results showed an effect on wall-sit performance [F = 10.64, *p <* 0.001]. The results of the Bonferroni showed a substantial increase in wall-sit performance in the HSB condition compared to BL [MD = 11.00, *P* = 0.004, 95% CI (3.44–18.55)] and PLA [MD = 8.75, *P* = 0.003, 95% CI (2.93–14.56)]; however, there was no difference between PLA and BL [MD = 2.25, *P* = 1.000, 95% CI (−4.62 – 9.12)] (Figure 4A). Also, the results revealed that the main effect on the 1RM leg press was significant [F = 9.48, *p* = 0.023]. The results of the *post hoc* test indicated that the 1RM leg press increased substantially in HSB compared to the PLA condition [MD = 6.56, *P* = 0.019, 95% CI (0.96–12.15)]; nevertheless, there were no differences between HSB and BL [MD = 4.06, *P* = 1.000, 95% CI (−7.22 – 15.34)] and PLA and BL [MD = −2.50, *P* = 1.000, 95% CI (−10.46 – 5.46)] (Figure 4B). However, there was no significant difference between the BL, and the HSB and PLA conditions in VJH [F = 0.98, *p* = 0.384], PPT [F = 2.01, *p* = 0.151], and VSFT [F = 0.17, *p* = 0.840], Figures 5A–C; Table 2). All of these results are reported in Table 3.

**TABLE 2:** Means and Standard deviation (SD) of the measured variables in the three conditions (n = 16).

Variables	BL	PLA	HSB
Wall-sit (s)	74.31 ± 5.76	76.56 ± 6.48	85.31 ± 6.67^¥^ ^a^
1RM leg press (Kg)	110.93 ± 30.17	108.43 ± 27.18	114.99 ± 27.14^a^
Vertical jump height (cm)	31.18 ± 6.51	30.68 ± 5.57	31.62 ± 5.64
PPT (mmHg)	127.68 ± 3.46	132.43 ± 3.09	134.62 ± 1.99
VSFT (cm)	30.50 ± 1.91	31.75 ± 2.46	31.20 ± 1.31

BL, baseline; PLA, placebo; HSB, honey-sweetened beverage; PPT, pressure pain threshold; VSFT, V sit and reach.

^a^ Significant difference compared to PLA (*P* < 0.05). ¥, Significant difference compared to BL (*P* < 0.05).

**TABLE 3:** Comparison of the variables data between three conditions.

		PLA (n = 16)		HSB (n = 16)	
		BL	HSB	BL	PLA
Wall-sit(s)	MD	2.250	−8.75	11	8.75
	Sig	1.000	0.003	0.004	0.003
	95% CI	−4.62–9.12	−14.56–−2.93	3.44–18.55	2.93–14.56
1RM leg press (Kg)	MD	−2.50	−6.56	4.06	6.56
	Sig	1.000	0.019	1.000	0.019
	95% CI	−10.46–5.46	−12.15–−0.96	−7.221–15.346	0.96–12.15
Vertical jump height (cm)	MD	−0.500	−0.937	0.438	0.937
	Sig	1.000	0.483	1.000	0.483
	95% CI	−1.84–0.84	−2.64–0.77	−1.78–2.66	−0.77–2.64
PPT (mmHg)	MD	4.75	−2.18	6.93	2.18
	Sig	0.338	1.000	0.318	1.000
	95% CI	−2.84–12.34	−11.97–7.6	−3.92–17.80	−7.6–11.97
VSFT (cm)	MD	1.25	0.554	0.703	−0.554
	Sig	1.000	1.000	1	1.000
	95% CI	−3.81–6.32	−6.58–7.68	3.97–5.37	−7.68–6.58

BL, baseline; PLA, placebo; HSB, honey-sweetened beverage; MD, mean difference; CI, confidence interval; PPT, pressure pain threshold; VSFT, V sit and reach.

## Discussion

This study aimed to determine the effects of HSB supplementation on performance recovery and muscle soreness symptoms following EIMD, including 1RM, endurance, flexibility of leg muscles, and vertical jump, as well as PPT and DOMS. The present study’s results illustrated that drinking a 250 mL honey-sweetened beverage 90 min before the EIMD significantly improved lower-body muscle endurance compared to the baseline and PLA, as well as increased 1RM of leg press and decreased RPE compared to PLA. Furthermore, the results indicated that HSB significantly reduced DOMS values immediately, 12, 24, and 48 h after EIMD compared to PLA. Nevertheless, there was no substantial difference in VJH, VSFT, and PPT. However, following the HSB intervention, VJH increased by 1.3% compared to the baseline (BL) and 2.9% compared to the PLA. Additionally, PPT showed an improvement of 5.1% compared to the BL and 1.6% compared to the PLA, and VSFT increased by 2.2% compared to the BL.

Honey is a natural food composed of several monos and disaccharides, mainly fructose (about 35%–40%), glucose (about 30%–35%), and other compounds such as water, polyphenols, vitamins, and minerals. Also, studies have shown that consuming each compound alone improves physical and physiological functions, so consuming honey with these multiple compounds has many health benefits (Hills et al., 2019). Honey consumption may theoretically have positive effects when consumed before, during, or after sports activity (Hills et al., 2019). As has been reported, honey consumption can improve physical performance at different levels of sports activities (Hills et al., 2019; Hatchett et al., 2016). This natural product can also protect the body from fatigue and inflammation following severe exercise, although physical function may not improve. The low GI and anti-fatigue properties of honey cause physiological adaptation to planned exercise among people with different physical conditions. However, several positive health effects may result from the synergistic interaction of honey and exercise therapy (Ali et al., 2021). It should be noted that the composition and amount of HSBs may play different roles in determining their effectiveness.

The results of the present research showed that drinking HSB leads to a reduction of DOMS immediately, 12, 24, and 48 h after EIMD compared to PLA. It is believed that one of the reasons honey can prevent muscle injuries during strenuous exercise is due to its high content of carbohydrates and vitamins, particularly B vitamins. These vitamins are especially effective in preventing damage to muscle cells after high-intensity and exhausting exercises. Therefore, consuming honey supplements and drinks can benefit athletes who want to prevent muscle injuries during strenuous exercise (Kim et al., 2016). Muscle structural damage and DOMS occur due to fatigue caused by exercise principles such as increasing intensity and duration. Intense and exhausting exercises can lead to fatigue, depletion of muscle glycogen, and increased lactic acid and lactate accumulation (Plowman and Smith, 2013). These processes can cause a decrease in pH levels in muscle cells, resulting in fatigue and, ultimately, DOMS (Plowman and Smith, 2013). Studies have shown that with their anti-fatigue, anti-pain, anti-inflammatory, and antioxidant properties, honey supplements and drinks can improve athlete performance by reducing fatigue indicators (Esmaeelzadeh et al., 2022). Furthermore, consuming honey in amounts of 20 g per day or 70 g 90 min before exercising can positively impact physiological functions. It can help reduce oxidative biomarkers like lipid peroxidation and lower intercellular inflammation by reducing TNF-α and IL-1β levels (Tartibian et al., 2016; Rahim et al., 2017). It can also reduce DNA damage by increasing antioxidant enzymes (catalase, superoxide dismutase), increasing T-lymphocyte cells, and ultimately improving the total antioxidant capacity (Tartibian et al., 2016; Rahim et al., 2017). Also, it has been found that honey consumption has a significant effect on sports performance, perception of fatigue, improvement of blood glucose concentration, and immune responses before, during, and after exercise (Yusof et al., 2018). When consumed routinely over several weeks, honey may reduce many of the immune system disturbances commonly associated with a moderate-to-vigorous exercise program (Yusof et al., 2018). Regarding the reduction of muscle structural damage, it has been reported in a study that intermittent training and consumption of thyme honey leads to an improvement in the glycemic profile and positive changes in the expression of cardiac and anti-apoptotic genes (Behaeen et al., 2022). On the one hand, it has been reported that consuming thyme honey after a circular resistance training session in young men does not affect inflammation and muscle damage (Abdi et al., 2021). On the other hand, the current study showed that honey consumption can reduce DOMS, which is evidence that it can reduce cellular structural damage in muscle tissue. However, there are conflicting results due to various factors such as the type of subjects, training conditions (type, intensity, duration, time, number of repetitions and sets), and recovery time. Additionally, the dosage of the supplement or the way it is taken with another substance can affect the reported results.

Our study found that drinking HSB improved lower-body muscle endurance, 1RM of leg press, and RPE. Also, the relative change (RC) between HSB and the baseline showed a 2.30% increase in flexibility performance and a 5.44% increase in PPT after HSB consumption. It is believed that the carbohydrates in honey and its other compounds play an important role in sports performance during sports activities (Ooi and Chen, 2023; Tang, 2022). It is consistent with the findings of a previous study that showed an increase in 1RM in individuals who consumed 240 mL raw milk honey solution (Hatchett et al., 2016). Furthermore, a study investigated the effects of 50 g of honey + 0.5 g of royal jelly + 0.5 g bee-pollen supplementation in running 1,500 m. It showed that the experimental group performed faster in running 1,500 m compared to the control group (Aly et al., 2019). These results suggest that consuming honey supplements or drinks can improve sports performance and related indicators. Although the effect of honey supplements on the variables of our study has not been directly measured, previous research has shown that honey supplementation and beverage can improve physiological factors and functions, such as glucose metabolism, antioxidant status of plasma, and selected physiological parameters, which can serve as an alternative ergogenic aid for athletes during training and racing (Sukri et al., 2022). Another study examined the effect of honey supplementation on physical recovery and dietary supplementation of basketball players and found that it can reduce fatigue and speed up physical recovery (Tang, 2022). In summary, the current study’s results demonstrate that HSB, when administered, reduces DOMS. Compared to PLA, this reduction is immediate and sustained at 12, 24, and 48 h after EIMD. The accompanying improvement in lower-body muscle endurance and 1RM of leg press in the HSB condition further underscores its impressive performance compared to the PLA condition. As honey, a natural substance comprised of ∼80% carbohydrates (primarily fructose and glucose), is known to possess antioxidant, antimicrobial, and anti-inflammatory properties, it could reduce the DOMS in the current study. This reduction, in turn, has a significant positive effect on the strength and muscular endurance of the participants, offering a promising avenue for further research and potential benefits in sports science, nutrition, and exercise physiology.

It should be noted that studies have yet to be conducted on how honey supplementation affects the factors of PPT and RPE. However, a study that looked into saffron’s analgesic and anti-inflammatory effects on preventing and treating DOMS and PPT showed that the percentage of pressure pain tolerance in the saffron group was higher and more significant than in the other groups. The researchers affirmed that saffron consumption is effective in preventing and treating pain and inflammation caused by DOMS (Meamarbashi and Rajabi, 2015). This could be attributed to the improvement of DOMS indicators following using antioxidant supplements such as honey and saffron. Additionally, DOMS causes membrane damage to some muscle strands, disrupting calcium homeostasis from extracellular sources and activating arachidonic acid metabolism (Meamarbashi and Rajabi, 2015). Arachidonic acid metabolism sensitizes the neural cords to chemical and mechanical movements and increases muscle pain perception. Therefore, it is possible that taking honey and saffron supplements can reduce muscle pain by improving calcium homeostasis mechanisms (Meamarbashi and Rajabi, 2015). The increase in 1RM in this study is likely due to the significant reduction of DOMS and increase in PPT resulting from consuming honey supplements (Tartibian et al., 2016). Also, our study showed that drinking HSB does not affect vertical jump height. This finding is inconsistent with a study that examined the consumption of honey and aerobic exercise in sedentary women and a study that examined the combination of honey consumption with exercise on the amount of long jump distance (Mohamed et al., 2021; Rahim et al., 2016). These variations are attributed to differences in the number of participants and the duration of honey supplement consumption. For instance, the Mohamed et al. (2021) study, with its 40 participants (16 in the current study) and the supplement consumption over 8 weeks (Rahim et al., 2016), presents a stark contrast to the current study’s acute consumption approach.

As previous studies have reported, skeletal muscle adapts to exercise-induced muscle damage in such a way that it is protected from subsequent damaging stimuli. This phenomenon is widely known as the repeated bout effect (Hyldahl et al., 2017). Additionally, the muscle force-generating ability shows the most significant decrement immediately after EIMD, with a linear force restoration during the next 7–14 days (Hyldahl et al., 2017). In contrast, DOMS develops at 1 day and peaks 2–3 days post-EIMD, then subsides and disappears up to 7 days after exercise (Nosaka and Tiidus, 2008). Also, the current study has two EIMD sessions with a one-week wash-out period. The results revealed improved muscle strength (1-RM) and DOMS after HSB ingestion. As the current study was conducted in a randomized, placebo-controlled, cross-over, and double-blind manner with a one-week wash-out, the repeated bout effect on the observations cannot be excluded.

The current study has several limitations that warrant attention. First, a notable limitation is the absence of measurements for biomarkers associated with muscle damage and soreness. Additionally, more objective parameters such as biochemical data (CK, LDH, and CRP) and the observation of glucose or insulin concentrations alongside supplementation could have enhanced the study findings. However, financial constraints precluded the fulfillment of these objectives. Secondly, the study did not evaluate cognitive indicators, arousal levels, or other psychological variables influencing muscle function. Furthermore, capturing additional baseline data after a one-week washout was unfeasible due to limited laboratory time and incomplete participant engagement. Given these limitations, it is imperative to exercise caution when interpreting this study’s findings. Consequently, future research endeavors should assess these factors to augment the breadth of our findings.

## Conclusion

In conclusion, our findings illustrated that drinking honey-sweetened beverages significantly improved lower-body muscle endurance compared to the baseline and PLA, as well as increased 1RM of leg press and decreased RPE compared to PLA. Furthermore, the results indicated that HSB significantly reduced DOMS values immediately, 12, 24, and 48 h after EIMD compared to PLA. Nevertheless, there was no significant difference in VJH, VSFT, and PPT. Additionally, according to the present study’s findings, it may be beneficial for strength-trained females to incorporate honey-based beverages before training sessions to improve some recovery indicators, such as muscle strength and endurance, local pain, and DOMS after EIMD. Also, future studies should focus on identifying the underlying mechanisms of HSB effects on the recovery of sports performance and DOMS.

## Data Availability

The original contributions presented in the study are included in the article/supplementary material, further inquiries can be directed to the corresponding author.
