# Corrigendum: Effect of a honey-sweetened beverage on muscle soreness and recovery of performance after exercise-induced muscle damage in strength-trained females

**DOI:** 10.3389/fphys.2024.1523903

**Published:** 2024-12-04

**Authors:** Hadis Hemmati, Walaa Jumah Alkasasbeh, Mohammad Hemmatinafar, Mohsen Salesi, Sepideh Pirmohammadi, Babak Imanian, Rasoul Rezaei

**Affiliations:** ^1^ Department of Sport Sciences, Faculty of Education and Psychology, Shiraz University, Shiraz, Iran; ^2^ Program of Sports Management and Training, Department of Administration and Curriculum, Faculty of Arts and Educational Sciences, Middle East University, Amman, Jordan

**Keywords:** honey-sweetened beverage, DOMS, recovery, female athletes, strength performance

In the published article, there was an error in **Affiliations** for Walaa Jumah Alkasasbeh. Instead of Walaa Jumah Alkasasbeh^2,3^, it should be “Walaa Jumah Alkasasbeh^2^.”

The authors apologize for this error and state that this does not change the scientific conclusions of the article in any way. The original article has been updated.

